# Load balancing prediction method of cloud storage based on analytic hierarchy process and hybrid hierarchical genetic algorithm

**DOI:** 10.1186/s40064-016-3619-x

**Published:** 2016-11-17

**Authors:** Xiuze Zhou, Fan Lin, Lvqing Yang, Jing Nie, Qian Tan, Wenhua Zeng, Nian Zhang

**Affiliations:** 1 Software School, Xiamen University, Xiamen, China; 2 Xiamen Institute of Software Technology, Xiamen, China

**Keywords:** Radial basis function neural network (RBFNN), Analytic hierarchy process (AHP), HHGA (hybrid hierarchical genetic algorithm), Load balancing, Cloud storage, Group decision

## Abstract

With the continuous expansion of the cloud computing platform scale and rapid growth of users and applications, how to efficiently use system resources to improve the overall performance of cloud computing has become a crucial issue. To address this issue, this paper proposes a method that uses an analytic hierarchy process group decision (AHPGD) to evaluate the load state of server nodes. Training was carried out by using a hybrid hierarchical genetic algorithm (HHGA) for optimizing a radial basis function neural network (RBFNN). The AHPGD makes the aggregative indicator of virtual machines in cloud, and become input parameters of predicted RBFNN. Also, this paper proposes a new dynamic load balancing scheduling algorithm combined with a weighted round-robin algorithm, which uses the predictive periodical load value of nodes based on AHPPGD and RBFNN optimized by HHGA, then calculates the corresponding weight values of nodes and makes constant updates. Meanwhile, it keeps the advantages and avoids the shortcomings of static weighted round-robin algorithm.

## Background

With the extensive applications of cloud computing, open-source projects of cloud computing platform construction such as Hadoop, Eucalyptus, CloudStack, and OpenStack are also increasing (Peng et al. 2009). For now, OpenStack (Pepple 2011) is one of the most popular open source projects (Sefraoui et al. 2012). Many large companies at home and abroad such as Intel, IBM, Cisco, and HP regard OpenStack as promising open source project. The main reasons include its fully open source, good design and high community vitality. OpenStack Swift (2012) is a storage system aiming at objects which have multi-tenant, powerful extensibility, redundancy and persistency, which can store large amounts of unstructured data through HTTP-based RESTful APIs at a low cost, and also has the advantage of no single point of failure. Using OpenStack Swift to construct cloud storage platform applications under production environments needs a load balancer to distribute access requests to Proxy Node (Pepple 2011). Therefore, load balancing is still needed for optimization.

Currently, load balancing can be classified from several different angles, including (1) static load balancing and dynamic load balancing, in which dynamic load balancing (Dhakal et al. 2007; Alakeel 2010) can determine the state of cluster nodes in real time and balance scheduling timely, thus improving the overall performance without adding too much computational overhead; (2) centralized and distributed load balancing. A common centralized load balancing cluster can achieve global optimal scheduling policy, while distributed load balancing is more adapted to complex situations of multiple rooms, multiple clusters and elastic expansion. Compared with the simple structure of centralized load balancing, the distributed load balancing strategy (Randles et al. 2010) often requires more communication overhead and network complexity; its scheduling strategy often achieves greater local optimal solutions. (3) The main differences of recipient, sender and symmetric start algorithm lie in which the starting point of load scheduling is between task requester and service provider (Waraich 2008). (4) Other categories also include: local and global load strategies (Waraich 2008; Khan et al. 2011), collaboration and non-cooperative load strategies (Waraich 2008; Yagoubi et al. 2006), adaptive load strategy and so on. In view of the characteristics of commercial computing services and management costs of cloud computing, this paper mainly studies how to realize the centralized, dynamic load balancing intelligent strategy.

Compared with static load balancing algorithm, the dynamic load balancing algorithm is of high complexity, because it needs to collect the extra overhead of load information, but it takes the current state of each service node in the cluster into consideration, and can give full play to the processing capacity of each service node to improve the throughput of cluster system. If allocation scheduling is proper, the overhead paid is necessary for improving the cluster system performance. Wherein the running state of service nodes is reflected through a variety of load information, and thus the load evaluation determines the merits of the request-allocation algorithm.

Typical methodology on load forecasting are BP neural network algorithm (Xiao et al. 2009) and the prediction algorithm based on filtering theory (Xu et al. 2000). However, it is very difficult to establish a common prediction method for all applications. Due to the differences of nature, different applications need corresponding and appropriate prediction methods for load predictions. Taking the virtual cluster OpenStack as the study object, this paper uses Proxy Node to monitor the load of Cloud computing nodes regularly, and puts forward that OpenStack cloud load prediction method of RBF neural networks combined with AHP and hybrid hierarchy genetic training. Simulation experiment results have shown that the prediction accuracy of the method is relatively good and it is feasible to provide services for the load balancing algorithm.

There are many the common load balancing algorithms in cloud computing environments: Round-Robin Scheduling (RR) (Hahne 1991), Weighted Round-Robin Scheduling (WRR) (Katevenis et al. 1991), Least-Connection Scheduling (LC) (He et al. 2015), Equally Spread Current Execution Load (ESCEL) (Tangang et al. 2014), Throttled load balancing algorithm (Throttled) (Tyagi and Kumar 2015) and Honeybee Foraging Algorithm (HFA) (Nakrani 2004). We introduce three of them briefly as follows:

1. Round-Robin Scheduling (RR)

RR Scheduling (Hahne 1991) is to distribute assignment requests sequentially to multiple cluster nodes, and that cycle repeats. RR Scheduling is simple and has high efficiency when the configurations and performance of hardware and software nodes are consistent, but when the cluster nodes have different performance and processing capacities, as RR Scheduling does not consider the load of each node, it is likely to cause load imbalance, thereby making the entire system perform poorly. RR Scheduling is adopted as the load balancing strategy for the Eucalyptus-based cloud computing platform (Shreedhar and Varghese 1996).

2. Equally Spread Current Execution Load (ESCEL)

ESCEL is a dynamic load balancing algorithm that requires a load balancer to monitor tasks to be addressed (Tangang et al. 2014). The function of the load balancer is to put the requested tasks into queue and assign them to different service nodes for processing. The load balancer frequently checks new tasks in the queue and then assigns them to a series of idle service nodes for treatment, while at the same time also maintaining the assignment list that has been assigned to be processed in the service node, which can help identify which services node is idle and new tasks can be assigned to it.

3. Throttled load balancing algorithm (Throttled)

The Throttled load balancing algorithm is entirely based on the virtual machine and it is a dynamic load balancing algorithm (Tyagi and Kumar 2015). In this algorithm, the user first requests the load balancer to find a suitable virtual machine to perform the task requested. In cloud computing of multiple virtual machine cases, according to the capabilities of the virtual machine to process assignment requests, first pre-assign a maximum number of user requests. When the requested tasks have reached the maximum number of the virtual machine, it will no longer continue to receive tasks.

## Load evaluation model based on AHP group decision making

Analytic hierarchy process (AHP) which is put forward by Saaty (Saaty 2003), is a tool for dealing with complex decisions and can help policy-makers to determine priorities and make optimal decisions. With such characteristics as systematic, flexible, simple, practical (Zhou and Wen 2014; Gass and Rapcsák 2004; Srdjevic 2005). AHP can be used for quantitative analyses of load evaluation of cloud storage, and calculates the load evaluation metric of the system by constructing a hierarchical structure model and comparative judgment matrix. Put forward the load evaluation based on the Group Decision Making AHP, by the combination with the characteristics of cloud computing environments, and the selection of information indicators which affect node load in the establishment of hierarchical structure, that including: CPU usage rate, internal storage usage rate, network I/O throughput, response time and so on. In the construction of the AHP comparison judgment matrix, the geometric mean method in group decision making is used, thereby reducing the subjective factor of man-made judgment matrix. It makes the load evaluation of service nodes in cloud computing environments more accurate.

### Establish hierarchical structure model

**Fig. 1:**
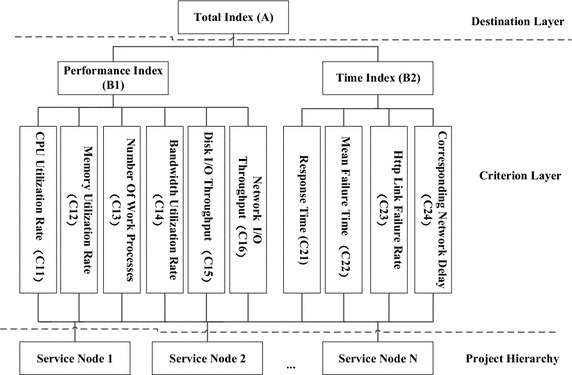
Hierarchical structure model of load evaluation

AHP requires dividing decision problems into the destination layer, criterion layer and project layer in accordance with their nature and affiliation to the establishment of a hierarchical structure. Among all levels, in order to avoid difficulties caused by the pairwise comparison of elements, the number of elements in the next layer dominated by a single element is generally no more than nine (Srdjevic 2005). Based on the study of the load of service nodes in cloud computing environments, we selected the load index which can accurately reflect the load condition of nodes to the maximum extent and followed the principles of integrity, measurability, dependence, simplicity and maneuverability of the cloud computing system. Eventually a hierarchical structure model for load evaluation of service nodes were established, which is shown in Fig. 1.

In Fig. 1, we present two first-grade indexes of the total load evaluation index: performance index (B1) and time index (B2). The performance index reflects the performance of nodes, which contains a number of common load information evaluation indexes—CPU utilization rate, memory utilization rate, number of work processes, and disk I/O throughput. Meanwhile, taking the importance of cloud computing network indexes into account, bandwidth utilization rate and network I/O throughput are included into node performance indexes. In time index series, response time, mean failure time, Http link failure rate and network response delay are selected since they all have a great relationship with the reliability of node service, and affect the quality of cloud storage service.

### Construct judgment matrix of pairwise comparison

Judgment matrix construction is a critical step of AHP, and after the establishment of hierarchical structure, the relationship between various elements (such as paralleling and affiliation) is determined. A judgment matrix is for a certain element of the upper layer, and evaluates the relative superiority degree between this layer and its associated elements. In order to have a certain degree of recognition and quantitative results, the general scale could be adopted to quantify the relative superiority degree of various elements, and thus a pairwise comparison judgment matrix will be constructed.

1 $$A = \left( {\begin{array}{*{20}l} 1 \hfill & {a_{12} } \hfill & \cdots \hfill & {a_{1n} } \hfill \\ {1/a_{12} } \hfill & 1 \hfill & \cdots \hfill & {a_{2n} } \hfill \\ \cdots \hfill & \cdots \hfill & \cdots \hfill & \cdots \hfill \\ {1/a_{1n} } \hfill & {1/a_{2n} } \hfill & \cdots \hfill & 1 \hfill \\ \end{array} } \right)$$

The comparison judgment matrix has the following three features:

1. $$a_{ij} > 0\left( {i,j = 1,2,3, \ldots ,n} \right);$$
2. $$a_{ij} = \frac{1}{{a_{ji} }};$$
3. $$a_{ii} = 1;$$

The scale values *a*_*ij*_ often take the reciprocals of the scales, and the meaning of each scale is shown in Table 1.

**Table 1:** Scale values and their meanings

Scale	Meaning
1	The two comparison elements are equally important
3	The former element is slightly more important than the latter
5	The former element is more important than the latter
7	The former element is much more important than the latter
9	The former element is definitely more important than the latter
2, 4, 6, 8	The important degree is between that of the above scales
Reciprocal	If the importance ratio of element i and element j is, then the importance ratio of element j and element i is

Since the integer and reciprocal between meet the mental habits of people when they are making judgment, and just as many psychology researches show that when comparing certain property of a set of things, and if being satisfied with the judgment, average people can generally and correctly identify the grade of property or the amount of things between (Benítez et al. 2015), thus choosing as the quantization of qualitative level are accepted and applied widely.

Here, we adopt the group decision-making method to construct a judgment matrix. First, people with relevant knowledge construct the judgment matrix of A, B1 and B2 separately and independently. Then, they adopt the geometric method which calculates corresponding elements of different judgment matrixes to construct the group decision making judgment matrix (Benítez et al. 2015) to meet the matrix consistency and reduce one-sidedness and subjectivity of individual construct matrixes. Suppose the judgment matrix person k construct is:

2 $$A^{\left( k \right)} = \left( {a_{ij} } \right)_{n \times n} = \left( {\begin{array}{*{20}l} 1 \hfill & {a_{12}^{\left( k \right)} } \hfill & \cdots \hfill & {a_{1n}^{\left( k \right)} } \hfill \\ {a_{12}^{\left( k \right)} } \hfill & 1 \hfill & \cdots \hfill & {a_{2n}^{\left( k \right)} } \hfill \\ \cdots \hfill & \cdots \hfill & \cdots \hfill & \cdots \hfill \\ {a_{n1}^{\left( k \right)} } \hfill & {a_{n2}^{\left( k \right)} } \hfill & \cdots \hfill & 1 \hfill \\ \end{array} } \right)$$

Wherein $a_{ij} = 1/a_{ji} ,k = 1,2, \ldots ,{\text{m}}$, $A^{\left( 1 \right)} ,A^{\left( 2 \right)} , \ldots ,A^{\left( m \right)}$ meets the order consistency.

Then make $${\bar{\text{A}}} = \left( {{\bar{\text{a}}}_{\text{ij}} } \right)_{{{\text{n}} \times {\text{n}}}}$$

The elements ${\bar{\text{a}}}_{\text{ij}} = \left( {\mathop \prod \limits_{{{\text{k}} = 1}}^{\text{m}} {\text{a}}_{\text{ij}}^{{\left( {\text{k}} \right)}} } \right)^{{\frac{1}{\text{m}}}}$ of the formula are made up of the ${\bar{\text{A}}}$ geometric mean of the corresponding elements of *m* judgment matrixes.

For the performance index *B1* in Fig. 1, the judgment matrix *B1* obtained by group decision-making is shown as follows:

3 $$B1 = \left[ {\begin{array}{*{20}l} {1 } \hfill & 2 \hfill & 3 \hfill & 5 \hfill & 3 \hfill & 5 \hfill \\ {1/ 2 } \hfill & 1 \hfill & 2 \hfill & 4 \hfill & 2 \hfill & 4 \hfill \\ {1/ 3} \hfill & {1/2} \hfill & 1 \hfill & 3 \hfill & 1 \hfill & 3 \hfill \\ {1/5} \hfill & {1/4} \hfill & {1/3} \hfill & 1 \hfill & {1/2 } \hfill & 1 \hfill \\ {1/3} \hfill & {1/2} \hfill & 1 \hfill & 2 \hfill & 1 \hfill & 2 \hfill \\ {1/5} \hfill & {1/4} \hfill & {1/3} \hfill & 1 \hfill & {1/2} \hfill & 1 \hfill \\ \end{array} } \right]$$

For the time index *B2*, the judgment matrix *B2* obtained by group decision-making is shown as follows:

4 $$B2 = \left[ {\begin{array}{*{20}l} 1 \hfill & {1/3} \hfill & {3 } \hfill & 2 \hfill \\ 3 \hfill & { 1} \hfill & {6 } \hfill & 5 \hfill \\ {1/3} \hfill & {1/6} \hfill & 1 \hfill & {1/2} \hfill \\ {1/2} \hfill & {1/5} \hfill & 2 \hfill & 1 \hfill \\ \end{array} } \right]$$

For the total index, the judgment matrix *A* obtained by group decision-making is shown as follows:

5 $$A = \left[ {\begin{array}{*{20}l} 1 \hfill & 3 \hfill \\ {1/3} \hfill & 1 \hfill \\ \end{array} } \right]$$

### Relative weight calculation and consistency check

In the analytic hierarchy process, the corresponding comparative judgment matrix of each criterion can be obtained by factors at their disposal. The single factor weight is obtained mainly through the ordering vector of solving matrix. Calculation steps are as follows:

1. Normalize the column vector of judgment matrix and get:

6 $$\tilde{A}_{ij} = \left( {\frac{{a_{ij} }}{{\mathop \sum \nolimits_{i = 1}^{n} a_{ij} }}} \right)$$

2. Get ${\tilde{\text{A}}}_{\text{ij}}$ according to the line

7 $$\overset{\lower0.5em\hbox{$\smash{\scriptscriptstyle\smile}$}}{W} = \left( {\sum\limits_{j = 1}^{n} {\frac{{a_{1j} }}{{\sum\nolimits_{i = 1}^{n} {a_{ij} } }}} ,\sum\limits_{j = 1}^{n} {\frac{{a_{2j} }}{{\mathop \sum \nolimits_{i = 1}^{n} a_{ij} }}, \ldots ,\sum\limits_{j = 1}^{n} {\frac{{a_{nj} }}{{\mathop \sum \nolimits_{i = 1}^{n} a_{ij} }}} } } \right)^{T} ;$$

3. $$\overset{\lower0.5em\hbox{$\smash{\scriptscriptstyle\smile}$}}{\text{W}}$$ Normalize and get $${\text{W}} = \left( {{\text{w}}_{1} ,{\text{w}}_{1} , \ldots ,{\text{w}}_{\text{n}} } \right)^{\text{T}}$$ ;
4. At last, get the eigenvalue $\lambda = \frac{1}{\text{n}}\sum\nolimits_{{{\text{i}} = 1}}^{\text{n}} {\frac{{\left( {\text{AW}} \right)_{\text{i}} }}{{{\text{w}}_{\text{i}} }}}$ of maximum ${\text{A}} .$

In order to avoid inconsistent judgments from impacting the feasibility of weight, and further ensure that the judgment meets the conformance requirements, we tested the consistency of the judgment matrix. When verifying its consistency, we introduce *CI* (Consistency Index) as a quantity standard of the consistency degree of the judgment matrix.

8 $${\text{CI}} = \frac{{\lambda_{\hbox{max} } - n}}{n - 1}$$

Equation (8) shows that the judgment matrix is completely consistent, and the larger the value is, the worse the consistency of the judgment matrix will be. Due to the complexity of objective things and the diversity of people’s understandings, and also the relationships between the one-sidedness properly produced in people’s understanding and the amount of factors that causes problems & the size of the problem (Yang et al. 2013; Kim et al. 2016), the standard of judging whether a matrix has consistency alone is not enough. Therefore, AHP introduces mean random consistency index, as shown in Table 2. The ratio of consistency index and mean random consistency index constitute the consistency ratio of judgment matrix, viz. At that time, general definition considers the consistency of judgment matrix as acceptable, or that the judgment matrix has no consistency, which needs policymakers to make appropriate adjustment and corrections of the judgment matrix.

**Table 2:** Mean random consistency index RI

n	1	2	3	4	5	6	7	8	9
RI	0	0	0.58	0.90	1.12	1.24	1.32	1.41	1.45

Calculating according to the steps on the basis of the judgment matrix obtained above, we can get the eigenvalue maximum through *B2*, thus the relative weight vectors (eigenvectors) and consistency index *CI* are shown as follows:

9 $$W_{B1} = \left( {0.3709,0.2380,0.1452,0.0607,0.1244,0.0607} \right)^{T} ;$$

10 $$CI = \frac{{\lambda_{{\hbox{max} \left( {B1} \right)}} }}{n - 1} = \frac{6.075 - 6}{6 - 1} = 0.0150;$$

From Table 2, we can know the value of the mean random consistency index *RI*, thereby knowing that Matrix *B1* satisfying the following consistency by the *CR* value:

11 $$CR = \frac{CI}{RI} = \frac{0.0150}{1.24} = 0.0122 < 0.1$$

We can get the eigenvalue maximum through *B2*, thus its relative weight vector and consistency index CI are shown as follows:

12 $$W_{B2} = \left( {0.2220,0.5743,0.0773,0.1264} \right)^{T} ;$$

13 $$CI = \frac{{\lambda_{{\hbox{max} \left( {B2} \right)}} }}{n - 1} = \frac{4.05 - 4}{4 - 1} = 0.0170;$$

From Table 2, we can know that the value of the mean random consistency index *RI*, thereby knowing Matrix *B2* satisfying the following consistency by the *CR* value:

14 $$CR = \frac{CI}{RI} = \frac{0.0170}{0.90} = 0.0189 < 0.1$$

We can get the eigenvalue maximum through *A*, thus its relative weight vector and consistency index *CI* are shown as follows:

15 $$W_{A} = \left( {0.7500,0.2500} \right)^{T} ;$$

16 $${\text{CI}} = \frac{{\lambda_{\hbox{max} \left( A \right)} }}{n - 1} = \frac{2.00 - 2}{2 - 1} = 0;$$

By *CI* = 0, we can get that matrix *A* has consistency.

### Calculate load evaluation metric value

Based on the relative weight of each factor, we assume the data vectors of performance index and time index collected at the service nodes are ${\text{a}} = \left( {{\text{c}}_{11} ,{\text{c}}_{12} ,{\text{c}}_{13} ,{\text{c}}_{14} ,{\text{c}}_{15} ,{\text{c}}_{16} } \right)$, ${\text{b}} = \left( {{\text{c}}_{21} ,{\text{c}}_{22} ,{\text{c}}_{23} ,{\text{c}}_{24} } \right);$ the formula of computing node load evaluation metric S is shown as follows:

17 $${\text{S}} = \left( {a \times W_{B1} ,b \times W_{B2} } \right) \times W_{A}$$

As we can see from the equation above, the larger the load evaluation metric *S* is, the heavier the node load will be. Conversely, the smaller the value of *S* is, the lighter the node load will be.

## Optimization of RBF neural networks using HHGA

Here, we combine AHP group decision making with RBF neural networks effectively to make RBF neural networks become a neural network with the experience of experts in related fields through training. Training data are from the load evaluation framework of AHP group decision making. When a new evaluation process proceeds, we can obtain a comprehensive evaluation value, thus effectively reduce the complexity of the computation process simply through modifying the trained and mature input parameter RBFNN (Kim et al. 2016; Hopfield 1982; Kohonen 1982). The hierarchy system established according to the above method of Analytic Hierarchy, obtains the load evaluation metric from the relative load information index data collected from service nodes and AHP Group Decision Making as RBF neural network’s data set, of which some is regarded as the training set, and the rest is as test set, to validate the load prediction role of RBF neural network.

The basis Gaussian function is expressed as:

18 $$T_{i} \left( \varvec{x} \right) = e^{{ - \parallel \varvec{x} - \varvec{c}_{\varvec{i}} \parallel^{2} /2\delta_{i}^{2} }} \quad i = 1,2, \ldots ,m$$

Wherein, ||x − *c*_*i*_|| represents Euclidean distance between x and *c*_*i*_; *T*_*i*_ *(x)* represents the output of the *ith* hidden layer node; x is the n-dimensional input vector; m is the number of hidden layer neurons; *c*_*i*_ is the basis function center; *σ*_*i*_ is the basis width of the ith hidden layer node. Each neuron node of hidden layer has a radial basis function center vector *c*_*i*_, this vector and the input sample “*x*” have the same dimension and $c_{i} = \left[ {c_{i1} ,c_{i2} , \ldots ,c_{im} } \right]^{T} ,\; i = 1,2, \ldots ,m$; if the hidden layer has “*m*” neurons, it has “*m*” radial basis function centers.

The output layer of RBF network is a linear combination of hidden layer node outputs, and the output expression is shown as follows:

19 $$Y_{k} = \mathop \sum \limits_{i = 1}^{m} w_{ik} T_{i} \left( x \right)$$

Wherein, *ω*_*i*_ is the weight from the *ith* hidden layer node to the output layer node, and *p* is the number of neurons at the output layer. In a RBF neural network, the output of hidden layer node represents the degree of departure of input sample “*X*” from radial basis function center “*C*_*i*_” of hidden layer node. The network input layer implements non-linear mapping and the output layer implements linear mapping.

**Fig. 2:**
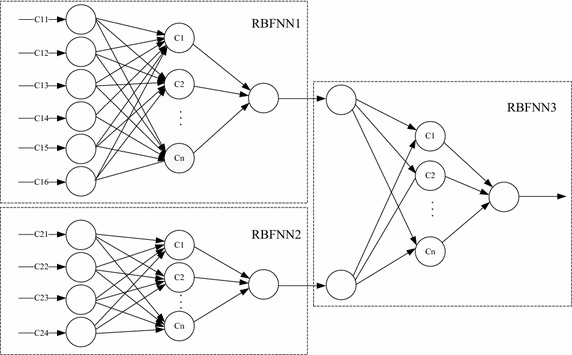
Load prediction model of RBF Neural Networks

The load prediction model based on RBF neural networks is shown as Fig. 2.

Previously, Sharifian and Motamedi compared the WRR, RBF and ANFIS on load balancing, the result show that RBF was better than others on convergence rate and prediction accuracy (Sharifian et al. 2011). Load prediction model based on HHGA-RBF neural network consists of three RBF neural networks, of which the RBFNN1 input in upper left corner is the data value of the load information evaluation index in the previous section, while the RBFNN2 input in lower left corner is $C_{21} ,C_{22} ,C_{23} ,C_{24}$, and these two RBF neural network outputs constitute the RBF input on the right and the final predicted value will be outputted by RBFNN3 on the right.

## HHGA optimizes RBF neural networks

A RBF neural network has a very good approximation performance. But in terms of design, there are two main difficulties. One is the parameters design, including: radial basis function’s center, width, and weight from hidden layer to output layer. Another is the numbers of nodes of the input layer and output layer are fixed, and the number and weight of the hidden layer nodes directly affect RBF performance. A common approach is to use the genetic algorithm to determine the node number, center and width of the hidden layer of RBF neural networks, which is a new study direction (Vesin and Grüter 1999; Billings and Zheng 1995; Gen et al. 2001) in recent years.

The hierarchical genetic algorithms (HGA) (Xing et al. 2011; Barreto et al. 2006) were put forward based on the hierarchy of biological chromosome which has two parts: controlling genes and parameter genes. The controlling genes which determine whether the parameter genes are activated, are expressed in binary form, in which “1” indicates that the underlayer genes are active, while “0” indicates that the underlayer genes are inactive. The hybrid coding method which combines binary coding with real number coding, takes the center, width, connection weights and topology of hidden layer nodes of RBF neural network as a whole and encodes them as a chromosome, and then selects the appropriate population size. The optimal results of number of the center, width, connection weight parameters and hidden node number of the hidden layer of RBF neural network are obtained through progressive optimization by genetic iterations. Although the hierarchical genetic algorithm is able to determine the parameters and structure of the RBF neural network, networking learning is slow in the convergence speed and efficiency.

This paper uses the Hybrid Hierarchy Genetic Algorithms (HHGA) to train RBF neural networks, namely to combine the HGA with the recursive least-squares method. The HGA can only determine the structure of RBF neural networks, the center and width of the hidden layer nodes, so this paper combines the HGA with the recursive least-squares method to construct connection weights between the hidden layer and the output layer. Determining the weights between the hidden layer and the output layer by the recursive least-squares method can ensure high convergence speed (Wang et al. 2012). Hybrid hierarchy genetic algorithm improves the efficiency of training the RBF neural network through hierarchical genetic algorithm, meanwhile retains the advantages of hierarchical genetic algorithm.

**Fig. 3:**
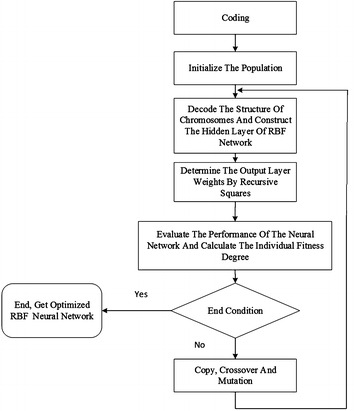
Processes of training RBF neural networks by HHGA

 Processes of training RBF neural networks by the HHGA are as shown in Fig. 3.

Processes of training RBF neural networks by the HHGA are as follows:

- Step 1 Code: Considering the parameters and its ability to find optimized solution of RBF neural network, genetic parameters are really encoded, and each gene is represented by a real number. The controlling genes are still adopts binary coding and each binary bit corresponds to the central coding and the width coding of a hidden layer node.
- Step 2 Generate initial population,
- Step 3 Decode individual, and construct the hidden layer of RBF neural networks.
- Step 4 Determine the weights by recursive squares

The exact weight solution is obtained by the recursion of the covariance matrix which is formed by the training sample input in each iteration. Error objective function is defined as follows:

20 $$E\left( n \right) = \frac{1}{2}\mathop \sum \limits_{k = 1}^{n} \lambda^{n - k} \mathop \sum \limits_{i = 1}^{M} \left( {d_{i} - y_{i} } \right)^{2}$$

Wherein formula (20) λ is the forgetting factor; *y*_*i*_ and *d*_*i*_ respectively represent the actual output and expected output.

Combining with the Eqs. (18) and (19):

$$T_{i} \left( \varvec{x} \right) = e^{{ - \parallel \varvec{x} - \varvec{c}_{\varvec{i}} \parallel^{2} /2\delta_{i}^{2} }} \quad i = 1,2, \ldots ,m$$

$$Y_{k} = \mathop \sum \limits_{i = 1}^{m} w_{ik} T_{i} \left( x \right)$$

The train data $\varvec{x}$, is the n-dimensional input vector. $c_{i} = \left[ {c_{i1} ,c_{i2} , \ldots ,c_{im} } \right]^{T}$ is the load evaluation index shown in Fig. 1. According to the Eq. (20), the error objective function $E\left( n \right)$, and the output of train data *y*_*i*_ and the expected output *d*_*i*_, refer to reference (Chen et al. 2011), training the connection weights of RBF neural networks, $w_{ik}$, by using the recursive least squares (RLS) method.

- Step 5 Evaluate the performance of RBF neural networks and calculate the individual fitness degree.

Considering that the objective of training RBF neural networks is to make it the simplest network structure while meet certain precision requirements, that is to make the approximation error precision and the complexity of the neural network in comprehensive index reach the minimum, in which the network approximation error precision objective function is represented by error sum of squares, and the network complexity is represented by the node number of the hidden layer, this paper adopts the fitness function as follows:

21 $$f = \frac{2N}{{\left( {a + be^{{\frac{M}{dn}}} } \right) \mathop \sum \nolimits_{i = 1}^{N} \left( {d_{i} - y_{i} } \right)^{2} }}.$$

In Eq. (21), *N* is the number of samples, *M* is the number of nodes in the hidden layer, n is the number of neural network input nodes, *y*_*i*_ is the network output corresponding to the *ith* input sample, *d*_*i*_ is the desired output, *a, b,* and *d* is constant.

- Step 6 Judge whether it meets the termination condition or not. If meet, then end it; otherwise, continue to the next step.
- Step 7 Select individual as parent based on individual fitness.

This paper adopts the selection operation which is based on the proportion of adaptation value, the probability of individual *i* being selected, where *f*_*i*_ is the fitness value of individual *i*, and denotes the sum of individual fitness values of the population.

- Step 8 The parent generation generates new individuals through cross and mutation, and parents are new individuals form new population.

In a hierarchical genetic strategy, it is necessary to do the crossover and mutation operations simultaneously with the controlling genes and parameter genes. The crossover probability and mutation probability adopt adaptive crossover and mutation probability. The crossover operation of the controlling genes follows the binary-coded cross rules. In order to make the real number coding of parameter genes produce new parameter genes through crossover operations, parameter genes generate a new parameter gene string by using a linear combination of the value of cross-bit corresponding to two parameter gene strings. The mutation operation of the controlling genes is a complementary operation of themselves with a certain probability. While the parameter genes follow an offset mutation, which is to add a random offset value to the mutation bit with a certain probability.

- Step 9 Return to Step 3 to continue.

## Dynamic load balancing algorithm implementation based on AHP and HHGA-RBFNN

Request tasks in a cloud computing environment are mutually independent. We use the HHGA-RBFNN model described above to predict the load of the current cloud computing system, and use the prediction result to calculate the polling weight of the cloud computing nodes periodically, and form a new dynamic load balancing algorithm—DPWRR through the combination with weighted round-robin algorithm.

### Basic principle of algorithm

The dynamic load balancing algorithm proposed here belongs to a centralized algorithm, which is to allocate the request tasks and deal with load balancing issues through a central node. When users issue a request, the load balancing policy of the central node decides which node to handle this task. The centralized load balancing algorithm mainly contains three steps:

1. The cloud computing load balancing center nodes predict the load of service nodes as per periodic time T;
2. Calculating a corresponding polling weight value for each back-end service node according to the prediction results by using HHGA-RBF model;
3. The central node allocates based on the polling weight value after receiving the request tasks.

After a period T, re-predict the load of nodes and calculate the corresponding polling weights. This method turns the static weight of the weighted polling algorithm to a dynamic adjustment one.

**Fig. 4:**
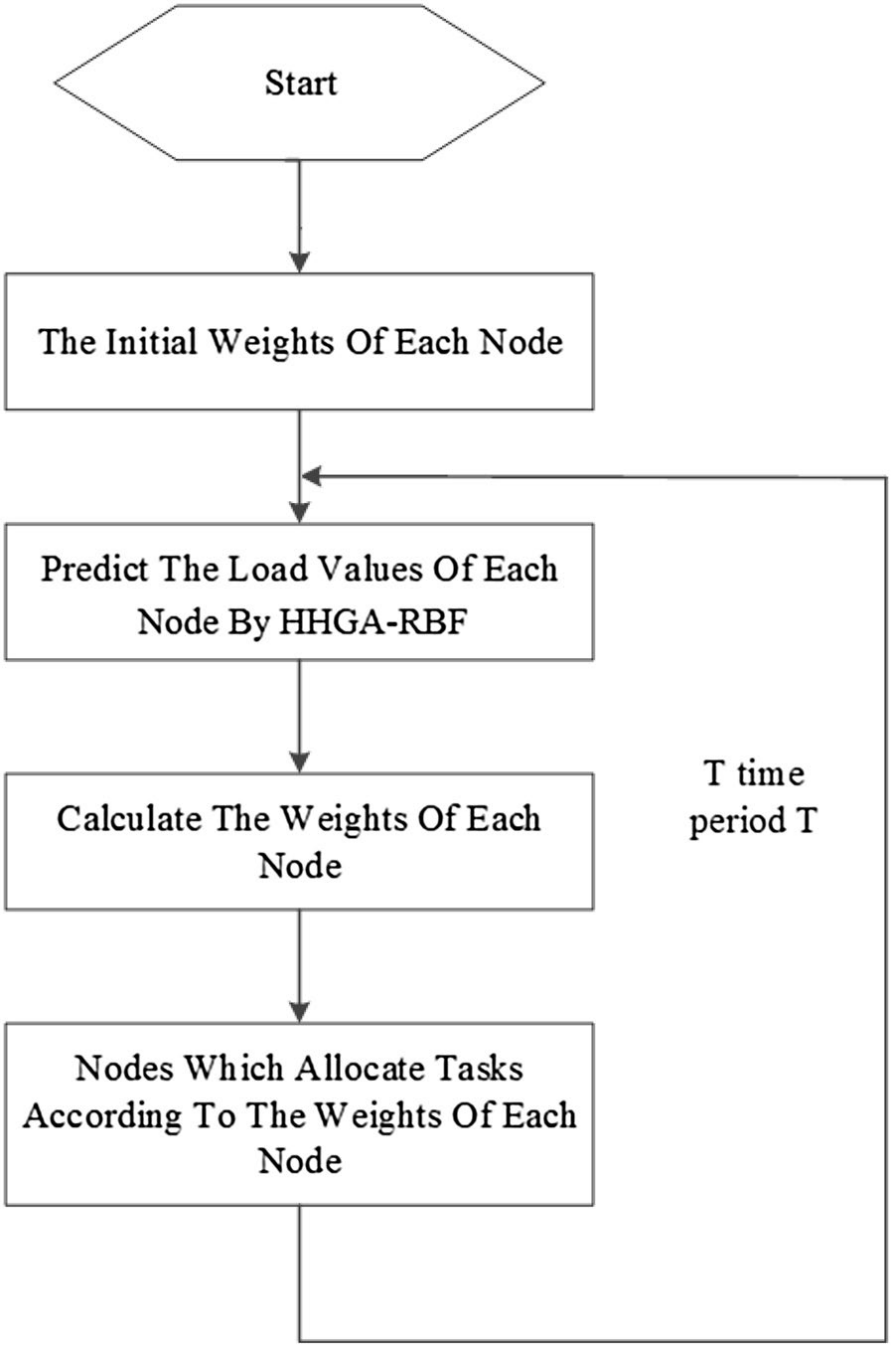
Processes of the load balancing algorithm

Processes of load balancing algorithm based on load prediction are shown in Fig. 4.

In an arithmetic process, the pseudo code of nodes which allocate tasks according to the weights of each node is shown in Algorithm 1.
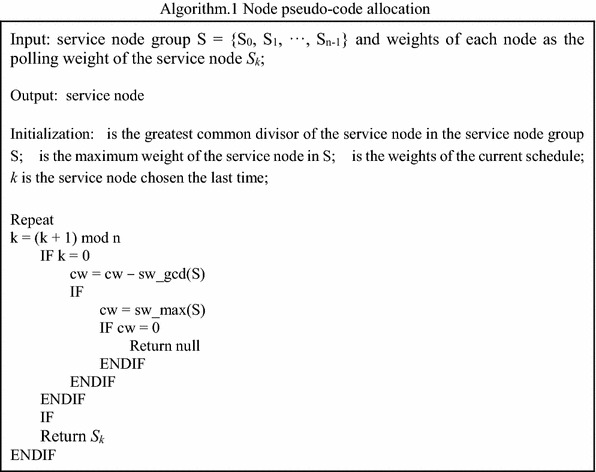


### Detail design

The information policy in this paper adopts the periodic collection methods and collects load index information on time, and the triggering policy is triggered by the central control node, that is to use the sender initiative. The algorithm proposed in this paper is focused on positioning strategy, and mainly considers the allocation of user request tasks, and it does not involve the migration strategy, thus involving no migration of virtual machines in cloud computing.

**Fig. 5:**
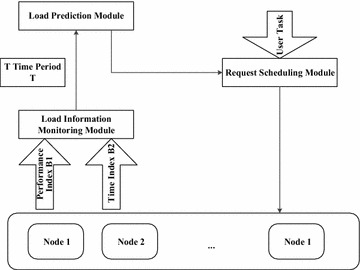
Algorithm block diagram

Several major modules of algorithm design is shown in Fig. 5.

1. Node load information monitoring module

In order to grasp the node load condition more accurately, solve the problem of node load prediction, and then calculate the polling weights of back-end service node according to the node load prediction results, we adopt the indexes chosen by the AHP group decision making and load evaluation method: performance index B1 and time index B2. The time period T which is related to the node load information collection is set in this module. And when the periodic time is reached, the node load information monitoring will be informed to collect the load information of the back-end node. Time period T must be set reasonably. Theoretically, the shorter the cycle time is, the more frequently the node load information collection will be, thus it is more able to reflect the node load condition, but if the node load information collection frequency is too high, it will cause unnecessary overhead and aggravation load. Generally, the collected time period T is set in 5–10 s (Drougas et al. 2006).

2. Load prediction module

Before performing load prediction, first we used group decision making AHP to make a load quantification of the index information collected by nodes. After collecting sufficient data, we can take them as the sample data to train the RBF neural network. Meanwhile we perform parameter optimization to RBF neural network with hierarchical genetic algorithm and recursive least square method. After obtaining the prediction model, the load prediction module predicts the load of data provided by the load information monitoring module.

3. Request scheduling module

We can first calculate the polling weights of the back-end service nodes and dynamically adjust the node weights as per the node load prediction value provided by the load prediction module, select the appropriate node to deal with tasks received following the weighted polling algorithm. The calculation formula of node polling weights is:

22 $$W_{i} = C \times \left( {{{1 - V_{i} } \mathord{\left/ {\vphantom {{1 - V_{i} } {\mathop \sum \limits_{i = 0}^{n} V_{i} }}} \right. \kern-0pt} {\mathop \sum \limits_{i = 0}^{n} V_{i} }}} \right)$$

where *W*_*i*_ is the weights of node *i*, *W*_*i*_ is the load prediction of node *i*, *C* adjusts constant in order to get an integer *W*_*i*_. When request scheduling module is in the initial state, all the polling weights of each node can be set as 1.

## Experiments

### Load prediction experiment of HHGA-RBF

**Fig. 6:**
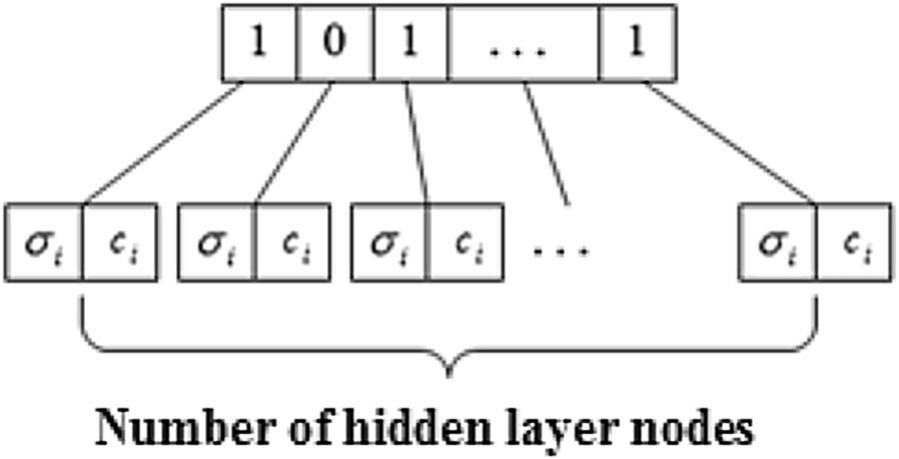
Hierarchical genetic algorithm for optimization of RBF hidden layer parameters

MATLAB software was used for doing a simulation experiment. Firstly, we can collect 1000 sets of data on the host of server, in which the parameter data under the performance indicators B1 as training samples to train RBFNN1 in the upper left corner of Fig. 6. Before network training, each class of sample parameter data is normalized.

The normalization formula is shown as follows:

23 $$y = \left( {x - MinValue} \right)/\left( {MaxValue - MinValue} \right)$$

In Eq. (23), $MaxValue$ is the maximum of such sample parameter data, and $MinValue$ is the minimum of such sample parameter data.

According to Qiang et al. (2011), we set the training parameters of RBF1 neural networks by the HHGA. Similarly, RBFNN2 should be trained. Finally, we can take the prediction value of RBFNN1 and RBFNN2 and input it in RBFNN3, and the server node load is predicted by RBFNN3.

**Fig. 7:**
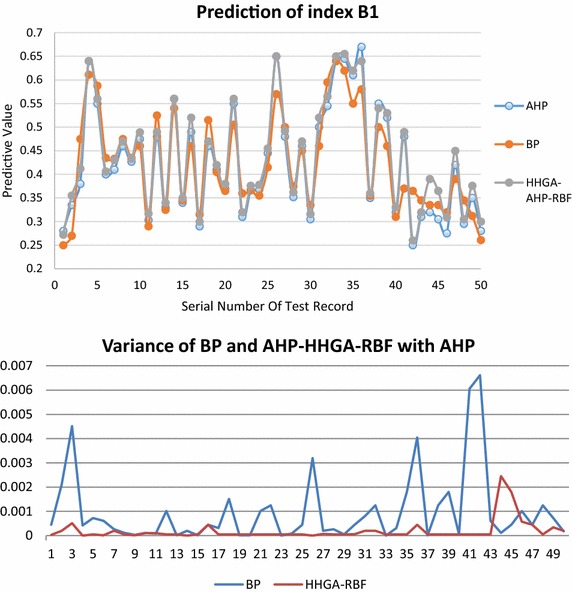
Prediction of index B1

**Fig. 8:**
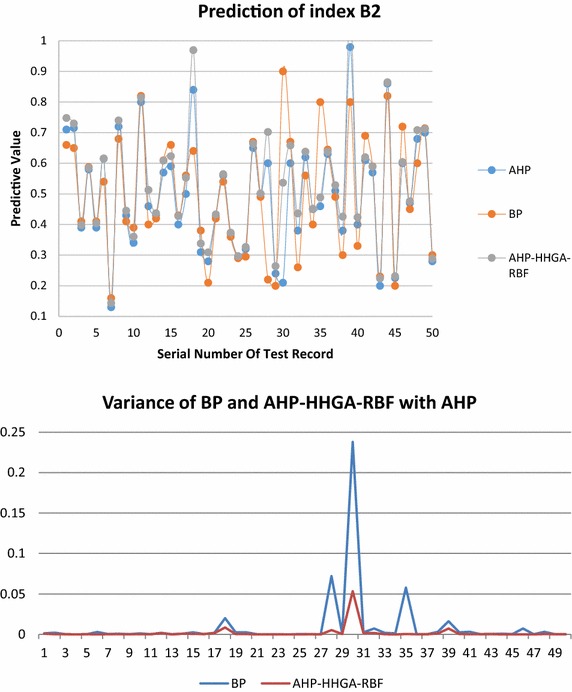
Prediction of index B2

Figures 7 and 8 are the prediction results comparison of index B1 and index B2 respectively, in which the HHGA-RBF prediction result is compared with the load evaluation value of AHP group decision making and BP prediction. The results show that the average relative error of RBF prediction values and AHP group decision making are less than 0.01, and are significantly better than the BP prediction results.

**Fig. 9:**
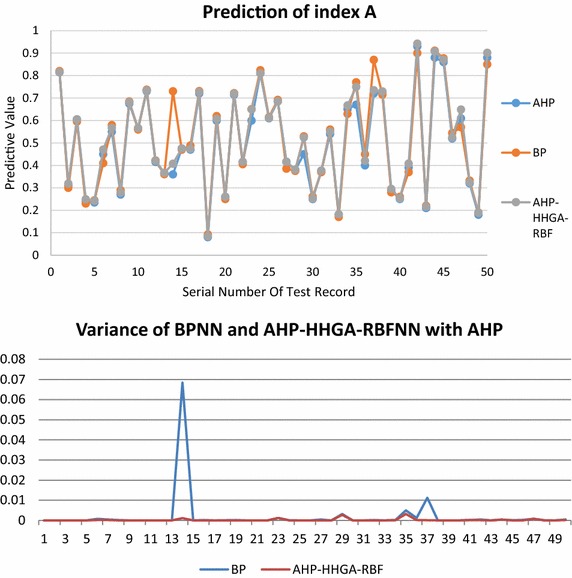
Prediction of total index A

The comparison of the prediction results of the total index A is shown in Fig. 9, which is the comparison of server node load predictions. The final server node load prediction uses HHGA-RBF prediction results, the average relative error is also less than 0.01 with the load evaluation value of AHP group decision making, and is also better than the prediction results of BP.

### Simulation environment configuration

In a CloudAnalyst simulation platform (Wickremasinghe et al. 2010), we configure user information, as shown in Table 3. Configurating user groups at different regional locations: UB1, UB2, UB3, UB4, UB5 and UB6; the cloud computing data center configuration is shown in Table 4. Here, we divides the cloud computing data center configuration into two kinds when testing the balancing algorithm performance: one is cloud computing data center CDC1 which is constituted by the data center DC1, and the other is the cloud computing data center CDC2 which is constituted by three data centers of DC1, DC2 and DC3; DC1 consists of 50 virtual machines which are virtualized by 10 physical hosts, DC2 consists of 35 virtual machines which are virtualized by eight physical hosts, DC3 consists of 25 virtual machines which are virtualized by 5 physical hosts.

**Table 3:** User group configuration

User base	Region	Requests per user per Hr	Data size per Request (bytes)	Peak hours (GMT)	Avg peak users	Avg off-peak users
UB1	0	90	200	3–5	3000	1000
UB2	1	50	200	5–7	2000	800
UB3	2	75	250	8–10	2800	1100
UB4	3	105	180	2–4	3500	1500
UB5	4	60	150	10–12	1500	600
UB6	5	80	200	3–9	2000	1000

**Table 4:** Data center configuration

Data center	Region	Arch	OS	VMM	Physical HW units	VMs
DC1	3	×86	Linux	Xen	10	50
DC2	3	×86	Linux	Xen	8	35
DC3	3	×86	Linux	Xen	5	25

The transmission delay and bandwidth between regional locations are shown in Tables 5 and 6, with a total of six regional locations.

**Table 5:** Transmission delay between regions, unit is ms

Region\region	0	1	2	3	4	5
0	25	100	150	250	250	100
1	100	25	250	500	350	200
2	150	250	25	150	150	200
3	250	500	150	25	500	500
4	250	350	150	500	25	500
5	100	200	200	500	500	25

**Table 6:** Bandwidth allocation between regions, unit is Mbps

Region\region	0	1	2	3	4	5
0	2000	1000	1000	1000	1000	1000
1	1000	800	1000	1000	1000	1000
2	1000	1000	2500	1000	1000	1000
3	1000	1000	1000	1500	1000	1000
4	1000	1000	1000	1000	500	1000
5	1000	1000	1000	1000	1000	2000

### Analysis of simulation results

In the cloud computing data center DC1 formed by CDC1, the simulation results of RR, ESCEL, Throttled and DPWRR load balancing algorithm proposed in this paper are shown in Tables 7 and 8. Table 7 is the overall response time of user groups under four different load balancing algorithms; Avg is the average response time of entire user groups of UB1, UB2, UB3, UB4, UB5 and UB6, Min is the minimum response time in the user groups, Max is the maximum response time in the user groups. Table 8 is the processing time of the data center.

**Table 7:** The overall response time of user groups under CDC1

Overall response time	Avg (ms)	Min (ms)	Max (ms)
RR	454.60	36.09	1300.27
ESCEL	454.47	35.84	1300.27
Throttled	454.47	35.84	1300.27
DPWRR	454.45	35.81	1300.24

**Table 8:** The processing time of data center under CDC1

Data center processing time	Avg (ms)	Min (ms)	Max (ms)
RR	0.64	0.09	11.21
ESCEL	0.50	0.09	2.01
Throttled	0.50	0.09	1.38
DPWRR	0.48	0.09	1.12

In the cloud computing data center formed by DC1, DC2 and DC3, the simulation results of RR, ESCEL, Throttled and DPWRR load balancing algorithm proposed in this paper are shown in Tables 9 and 10.

**Table 9:** The overall response time of user groups under CDC2

Overall response time	Avg (ms)	Min (ms)	Max (ms)
RR	485.83	36.74	1265.30
ESCEL	485.78	36.71	1265.30
Throttled	485.77	36.74	1265.30
DPWRR	485.77	36.69	1265.30

**Table 10:** The processing time of data center under CDC1

Data center processing time	Avg (ms)	Min (ms)	Max (ms)
RR	0.49	0.09	2.17
ESCEL	0.44	0.10	1.61
Throttled	0.44	0.09	1.14
DPWRR	0.44	0.09	1.29

**Fig. 10:**
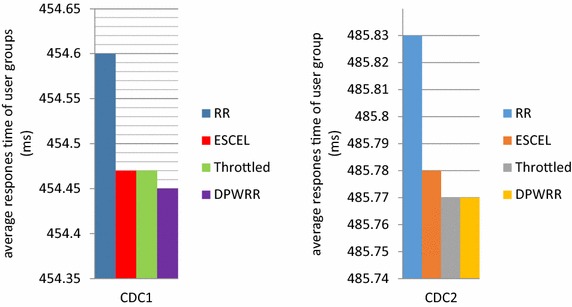
Comparison of average response time of user groups under CDC1 and CDC2 among the algorithm

The comparison of the average response time of user groups between the load balancing algorithm DPWRR designed by this paper and RR, ESCEL, Throttled under different configurations of CDC1 and CDC2 is shown in Fig. 10. In cloud computing data center CDC1, DPWRR is slightly better than the other three kinds of load balancing algorithms, as it has the minimum average response time in the user groups; in CDC2, the effects of DPWRR and Throttled are almost the same, but they are better than the RR and ESCEL load balancing algorithms.

**Fig. 11:**
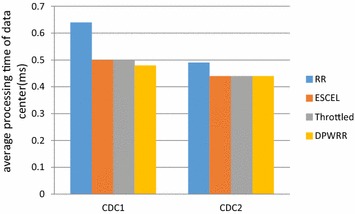
Comparison of average processing time of data centers under CDC1 and CDC2 among the algorithm

The comparison of the average processing time of data center among the four kinds of load balancing algorithms—DPWRR, RR, ESCEL and Throttled under cloud computing data centers of CDC1 and CDC2 is shown in Fig. 11. In the cloud computing data center CDC1, DPWRR is slightly better than the other three load balancing algorithms, as it has the minimum average processing time of all data centers; in CDC2, the average processing time of DPWRR, ESCEL and Throttled load balancing algorithms is almost the same, but slightly better than that of RR.

## Conclusion

This paper presents a dynamic load algorithm based on AHPGD and HHGA-RBF neural networks under the cloud computing environment, in order to solve the problem of load balancing of the allocation of user request tasks in cloud computing environment. It describes the basic idea and design of the algorithm based on the load prediction model, that constructed in the load prediction process uses a HGA and the recursive least-squares method to train parameters of RBF neural networks. The algorithm combines with weighted round-robin algorithm and dynamically updates the weights of each node within the time period. In the design details of the algorithm, we propose three modules of the algorithm: node load information monitoring module, load prediction module and request scheduling module; meanwhile this paper also designs the time period T and the calculation method of node weights. Finally, we further describe the simulation experiments of the proposed algorithm conducted with CloudAnalyst, and analyze its comparison with the algorithms of RR, ESCEL and Throttled, the results of which show that the algorithm is slightly better than the other three load balancing algorithms, which is effective and feasible.
